# Correlates of perceived health related quality of life in obese, overweight and normal weight older adults: an observational study

**DOI:** 10.1186/1471-2458-14-35

**Published:** 2014-01-15

**Authors:** Cinzia Giuli, Roberta Papa, Roberta Bevilacqua, Elisa Felici, Cristina Gagliardi, Fiorella Marcellini, Marco Boscaro, Marco De Robertis, Eugenio Mocchegiani, Emanuela Faloia, Giacomo Tirabassi

**Affiliations:** 1 Unit of Geriatrics, INRCA (Italian National Institute on Aging), Fermo, Italy; 2 Centre of Socio-economic Gerontological Research, Scientific-Technological Area, INRCA (Italian National Institute on Aging), Ancona, Italy; 3 Scientific Direction, INRCA (Italian National Institute on Aging), Ancona, Italy; 4 Unit of Rehabilitation Medicine, Scientific-Technological Area, INRCA (Italian National Institute on Aging), Ancona, Italy; 5 Division of Endocrinology, Department of Clinical and Molecular Sciences, Umberto I Hospital, Polytechnic University of Marche, Ancona, Italy; 6 Laboratory of Nutrigenomic and Immunosenescence, Scientific-Technological Area, INRCA (Italian National Institute on Ageing), Ancona, Italy

**Keywords:** Obesity, Older adults, Health related quality of life

## Abstract

**Background:**

Obesity is a complex multifactorial disease, which also has an impact on quality of life. The aim of this paper is to identify the correlates of perceived health related quality of life in obese, overweight and normal weight Italians older adults.

**Methods:**

205 subjects at the age ≥ 60 yrs. were recruited into the Division of Endocrinology of the Polytechnic University of Marche Region, Ancona (Italy). A protocol of questionnaires was constructed for data collection, and included domains such as physical activity, quality of life, socio-psychological aspects. The association of the latter variables with SF-36 Health Survey physical component (PCS-36) were evaluated in the whole sample. Multiple linear regression models were used to assess the effect of independent variables on PCS-36 and the physical subscales of SF-36.

**Results:**

PCS-36 showed a lower score in the obese and overweight subjects than the normal weight group (post-hoc test, p < 0.001 and p < 0.05 respectively). Age, gender (male), Body Mass Index, years of education, Physical Activity Scale for the Elderly (PASE) total score, Hospital Anxiety and Depression Scale anxiety, Hospital Anxiety and Depression Scale depression, number of medications prescribed and number of diseases were included in the model. Negative and significant PCS-associated variables included depression (p = 0.009), BMI (p = 0.001), age in years (p = 0.007), whereas positive and significant PCS-associated independent variables were years of education (p = 0.022), physical activity (p = 0.026). BMI was negatively associated with all the physical subscales of SF-36 (p < 0.05).

**Conclusions:**

Research funding should be invested in the study of the benefits accruing from reducing obesity in the elderly.

## Background

Obesity is considered an important health problem in many developed and developing countries. In 2008, overweight and obesity were estimated to afflict nearly 1.5 billion adults worldwide [1]. This phenomenon has been increasing rapidly in the last few decades in USA and many European countries, including Italy [2]. Indeed, some authors found that more than 50% of Italian adult and older men and about 1 of 3 women are overweight or obese [3].

Elevated morbidity and mortality in overweight and obese subjects is caused by an array of associated diseases which place a major public health burden on society [4], and include hypertension, coronary heart disease [5], type 2 diabetes [6], and cancer [7]. Further, these conditions are becoming increasingly prevalent in the elderly [8]. Metabolic and genetic factors among many others underpin this association, as well as obesity-related complications [9].

Indeed, some authors describe obesity as a complex multifactorial disease, which also has an impact on physical function and quality of life [10,11].

A study concluded that persons with obesity had significantly lower HRQL than those who were normal weight and such lower scores were seen even for persons without chronic diseases known to be linked to obesity [12].

Moreover, some authors appointed that obese older adults reported impaired quality of life in comparison with normal-weight people. In particular, they evidenced worse results on physical functioning and physical well-being [13]. These results reinforce the importance of normal body weight in older age.

Psychological problems are a common feature of obesity [14]. A recent study identified the strong association between depression, obesity and disability among the middle-aged, particularly in women [15]. Other psychological disorders, such as anxiety are associated with changes in body weight [16].

Moreover, a mix of socioeconomic, demographic and lifestyle factors as well as individual attitudes contribute to the risk for disease and obesity in older adults [17].

Many studies report the association between obesity and worsening health related quality of life, in both sexes [18,19].

Perceived health is a highly significant indicator in self-rated health status [20]. A study points out that in the domain of public health practice and research, perceived and self-rated health provide valuable insight into subjective health status thanks to the simplicity involved in gathering such data [21]. As such, this indicator is widely adopted and is acknowledged as a valid benchmark and independent predictor of mortality together with a variety of diseases and conditions, such as obesity [22].

Given this background, the aim of this paper is to analyze the correlates of health related quality of life (HRQL) in obese, overweight and normal weight Italians older adults.

## Methods

### Sample and recruitment

The subjects included in the study were selected from the patients attending the Division of Endocrinology, Department of Clinical and Molecular Sciences of Polytechnic University of Marche. The sampling method consisted in the selection of a consecutive series of subjects on the basis of specific inclusion/exclusion criteria, in a period of three years (January 2010–December 2012).

The study was designed to detect a mean difference between the 3 groups (normal weight, overweight and obese subjects) in the SF-36 Physical component summary score. Sample size was computed on the basis of preliminary results obtained from 30 subjects (10 each group) using G-Power version 3.1.3. Alpha level was placed at 0.05, while power was set at 90%. This produced a sample size of 144 patients (48 subjects each group).

Inclusion criterion was to be aged 60 years and over. Exclusion criteria were: 1) pituitary, thyroid, adrenal and gonad disorders not controlled by the ongoing therapy; 2) evident electrolyte disorders; 3) poorly controlled diabetes mellitus (glycated haemoglobin > 8%).

On the same day of enrolment, subjects were clinically and anamnestically assessed and, then, were asked to fill out the administered questionnaires.

A total of 433 subjects aged 60 years and over were screened: among these, 115 subjects did not give their consent to participate, while another 97 met the exclusion criteria. Sixteen subjects were excluded because inaccurately filled out the questionnaires (more than 50% of missing answers). Finally, 205 subject were included in the study.

The Ethics Committee of Polytechnic University of Marche approved the project and an informed consent was obtained from each individual in compliance with Italian legislation and the Helsinki Declaration.

### Instruments

#### Anthropometric measures and clinical data

A clinical evaluation and an extensive case-history assessment was performed for each patient, in order to collect clinical data relevant to our study, i.e. body Mass Index (BMI), waist and hip circumference, and to evaluate comorbidities and medication prescribed. Body Mass Index (BMI), waist and hip circumference were measured according to standard protocol: BMI was calculated as weight in kilograms divided by the square of the height in metres (kg/m^2^), and the World Health Organization classification of BMI for adult population was applied: normal weight, 18.5 ≤ BMI < 25.0 kg/m^2^; overweight, 25.0 ≤ BMI < 30.0 kg/m^2^; obesity, BMI ≥ 30.0 kg/m^2^[23].

Smoking habit was also asked, classifying the subjects as current smoker, ex-smoker or no smoker.

An aggregate score of the number of medical conditions, including cardiovascular, endocrine, metabolic, neurological and gastrointestinal diseases, was calculated for each respondent.

#### Socio-demographic characteristics

A protocol of questionnaires was constructed for the collection of socio-demographic data. Marital status (single, married, separated/divorced, widowed), kind of household (living alone/with other persons), educational level, years of education, and employment status (working/retired) are included within the scope of this paper.

Educational level was indicated as following:

1. Primary education, including subjects with primary school certificate and junior high school certificate, literate subjects but no school certificate;

2. Secondary education, including subjects with medium and high educational level, such as senior high school certificate;

3. Tertiary education, including subjects with high educational level, such as university degree.

#### Physical activity

The validated Italian version of Physical Activity Scale for the Elderly [24,25] was used to assess the amount of physical activity. The PASE consists of questions on self-reported occupational, household, and leisure activities over a one-week period. For each activity, the frequency (No. of times per week) and the duration (No. of hours) were asked. Total scores were calculated by multiplying the time spent on that activity for a specific weight and then adding up all the scores thereby obtaining a range of 0-400.

#### Quality of life

The Health-Related Quality of Life Scale (Short Form 36-item Health Survey) was used to assess quality of life [26]. The SF-36 measures diverse attributes of functional health status: physical functioning, role limitations due to poor physical health problems, bodily pain, general health, vitality (energy and fatigue), social functioning, role limitations arising from emotional problems and mental health (psychological distress and well-being). For each dimension, item scores are coded, summed, and transformed on to a scale from 0 (worst health) to 100 (best health) [27]. In addition, the SF-36 assesses overall physical and mental function using summary scales which include the Physical Component Summary Score (PCS-36) and Mental Component Summary Score (MCS-36). This instrument is reliable, valid and suitable for elderly people [28].

#### Anxiety and depression

The Hospital Anxiety and Depression Scale (HADS) [29] was used to assess levels of anxiety and depression. This instrument comprises two 7-item scales, one to evaluate anxiety and the other to assess depression. For each statement, the patient was asked to indicate which of four possible options best described his/her emotional state. The normative data classify scores less than or equal to 7 as normal, from 8 to 10 as borderline bases (situations that could potentially degenerate into anxiety or depression), and lastly scores above 11 indicating clinically relevant anxiety or depression as. The questionnaire is generally self-administered by the patient. The Italian version of this instrument is reliable, valid and suitable also for elderly people [30].

#### Social support

Social networks and informal social support were measured using the Lubben Social Network Scale (LSNS) which was specifically developed for use in older adults in both research and clinical settings (hospitals, nursing homes, clinics, day hospitals) [31]. The scale assesses the extent of social contact with family and friends. The total score is the sum of the items and ranges from 0 to 60, where high scores indicate good informal social support. This scale was previously used in another analysis to study the relationship between social support and quality of life in elderly [32].

#### Statistical analysis

Normality distribution of data was verified with the Kolmogorov-Smirnov test. Continuous data are expressed as mean ± SD, while categorical data as percentages. A descriptive analysis was performed, to evaluate the distribution of the variables in the three BMI classes (normal weight, overweight, obesity). Comparisons of mean values among three groups were made by one-way ANOVA followed by Bonferroni post-hoc test, while frequencies were compared using chi-square test. Pearson’s analysis was used to assess correlations among PCS-36 and MCS-36 and continuous variables in the total sample. ANOVA or t-test was used to compare PCS-36 mean values among categorical variables.

Lastly, the variables significantly associated with Physical component of SF-36 were included in different multiple linear regression models using PCS-36 and the physical subscales (Physical functioning, Role-physical, Bodily pain, General health) as dependent variables. All independent variables were included simultaneously in the regression model (Enter method). Independence of residuals and multicollinearity were verified by Durbin Watson and Variance Inflation Factor (VIF) statistic, respectively.

A value of p < 0.05 was accepted as statistically significant. Analyses were carried out using SPSS 16.0 statistical software for Windows (SPSS; Chicago, IL, 2002).

## Results

### Sample characteristics

Participants were predominantly female (73.6%) aged on average 68.7 ± 6.4 years (mean ± SD). Table 1 shows the main psychosocial and lifestyle characteristics of the subjects.

**Table 1:** Characteristics of the participants by BMI classes

	**Normal weight**	**Overweight**	**Obesity**	**p-value**
	**n = 86**	**n = 53**	**n = 66**	
Age (years)	69.02 ± 6.61	69.46 ± 5.69	67.76 ± 6.59	0.304
Gender				0.627
Male	25.6%	22.6%	30.3%	
Female	74.4%	77.4%	69.7%	
Waist circumference (cm)	82.99 ± 9.32	92.69 ± 8.36^a^	111.22 ± 12.47^ab^	**<0.001**
Hip circumference (cm)	97.16 ± 6.49	106.17 ± 6.48^a^	118.85 ± 12.15^ab^	**<0.001**
Number of medications prescribed	2.09 ± 1.85	2.36 ± 1.6	2.63 ± 1.97	0.198
Number of diseases	1.76 ± 1.39	1.77 ± 1	2.22 ± 1.58	0.089
Cardiovascolar diseases (yes)	44.2%	60.8%	66.7%	**0.015**
Endocrine and metabolic diseases (yes)	67.4%	78.4%	74.2%	0.350
Neurological and psychiatric diseases (yes)	2.3%	3.9%	6.1%	0.503
Gastrointestinal diseases (yes)	3.5%	2.0%	6.1%	0.507
Smoking habits				0.603
Not smoker	83.7%	86.8%	77.3%	
Ex-smoker	10.5%	5.7%	13.6%	
Smoker	5.8%	7.5%	9.1%	
Marital status				0.165
Single	4.9%	0.0%	4.7%	
Married	80.5%	86.3%	68.8%	
Divorced/separated	4.9%	3.9%	3.1%	
Widowed	9.8%	9.8%	23.4%	
Living alone (yes)	11.0%	9.8%	23.1%	0.063
Level of education				0.450
No education	1.2%	0.0%	3.1%	
Primary	54.9%	68.6%	64.6%	
Secondary	32.9%	25.5%	21.5%	
Tertiary	11.0%	5.9%	10.8%	
PASE (total score)	111.91 ± 50.39	114.94 ± 46.1	102.3 ± 55.37	0.218
Self-evaluation of health status				**0.044**
Excellent/very good	8.5%	6.0%	1.6%	
Good	43.9%	36.0%	26.6%	
Fair/poor	47.6%	58.0%	71.9%	
SF-36 Physical functioning	73.66 ± 27.76	68.1 ± 21.73	56.41 ± 26.75^a^	**<0.001**
SF-36 Role-physical	64.51 ± 39.9	49.0 ± 41.33	48.05 ± 42.56	0.053
SF-36 Bodily pain	63.17 ± 24.18	50.64 ± 25.37^c^	49.12 ± 23.39^d^	**0.001**
SF-36 General health	54.83 ± 19.15	48.96 ± 20.93	45.25 ± 18.64^c^	**0.013**
SF-36 Vitality	55.71 ± 17.53	50.40 ± 23.88	47.38 ± 18.07^c^	**0.036**
SF-36 Social functioning	70.43 ± 20.37	66.50 ± 28.06	62.69 ± 22.48	0.135
SF-36 Role-emotional	71.54 ± 37.81	65.33 ± 42.57	54.69 ± 42.98^c^	**0.048**
SF-36 Mental health	63.0 ± 16.23	62.40 ± 22.74	57.90 ± 19.30	0.248
SF-36 Physical component summary PCS-36	44.57 ± 10.75	39.75 ± 8.96^c^	38.19 ± 9.07^a^	**<0.001**
SF-36 Mental component summary- MCS-36	45.86 ± 9.03	45.33 ± 12.78	43.28 ± 10.13	0.321
LUBBEN SCALE (total score)	31.83 ± 9.08	33.16 ± 9.51	31.09 ± 10.62	0.520
HADS anxiety (total score)	6.34 ± 3.52	6.37 ± 4.05	7.22 ± 3.32	0.283
HADS depression (total score)	6.68 ± 3.7	7.45 ± 4.39	7.73 ± 3.85	0.265

`Note: Continuous variables are expressed as mean ± standard deviation; categorical data as percentage. Chi-square test or one-way ANOVA as appropriate; Bonferroni post-hoc test following ANOVA:

^a^p < 0.001 vs Normal weight.

^b^p < 0.001 vs Overweight.

^c^p < 0.05 vs Normal weight.

^d^p < 0.01 vs Normal weight.

42% of the subjects were in the normal weight range (BMI 18.5-24.9 Kg/m^2^), while 26% were overweight (BMI 25-29.9 Kg/m^2^) and 32% were obese (BMI > 29.9 Kg/m^2^). Among the groups, the percentage of smokers was higher in obese subjects (9.1%), though not significantly so. The majority of the subjects were retirees (82.5%), and as regards education, 61.6% had received primary schooling and 27.3% secondary schooling, with no significant differences among the groups. Even though there was no significant difference in the levels, the average anxiety and depression scores for the obese subjects were higher than in the other two groups.

Self-rated health status differed considerably among the three groups, as shown in Table 1.

Physical Component of SF-36 showed a lower score in the obese and overweight subjects than the normal weight group (post-hoc test, p < 0.001 and p < 0.05 respectively), while there were no significant differences for the Mental component (MCS-36). Additionally, the obese subjects also displayed lower scores for some of the SF-36 sub-scales, such as Physical functioning, Bodily pain, General Health, Vitality and Role-emotional.

### Correlation and multiple linear regression analysis

A correlation analysis was then performed to evaluate the variables associated with PCS-36 and MCS-36 (Table 2). MCS-36 was found to be not associated with BMI. For this reason, only PCS-36 was considered for further analysis. Categorical variables such as marital status, smoking habits, kind of household and presence of diseases did not show significant differences in PCS-36 score (data not shown).

**Table 2:** Pearson’s correlations between PCS-36 and MCS-36 and continuous variables

	**PCS-36**		**MCS-36**	
	**r**	**p-value**	**r**	**p-value**
BMI	−0.224	**0.002**	−0.079	0.276
Waist circumference (cm)	−0.257	**< 0.001**	−0.090	0.230
Hip circumference (cm)	−0.283	**< 0.001**	−0.084	0.273
Age in years	−0.246	**0.001**	0.068	0.343
Years of education	0.268	**< 0.001**	0.005	0.943
PASE total score	0.223	**0.002**	0.113	0.120
LUBBEN SCALE (total score)	0.029	0.685	0.214	**0.003**
HADS anxiety	−0.302	**< 0.001**	−0.656	**< 0.001**
HADS depression	−0.382	**< 0.001**	−0.538	**< 0.001**
N. of medications	−0.195	**0.006**	0.034	0.635
N. of diseases	−0.162	**0.025**	0.034	0.641

The following variables, significantly correlated with PCS-36, together with gender (male), were included in a multiple linear regression model as independent variables: age, BMI, years of education, PASE total score (physical activity), HADS anxiety, HADS depression, number of medications prescribed and number of diseases. The model was significant (F test = 8.840, p < 0.001) and produced a R-square of 0.291 (Table 3). Independent variables negatively and significantly associated with PCS-36 in the model were depression (p = 0.009), BMI (p = 0.001), age in years (p = 0.007). Conversely, the ones positively and significantly associated with PCS-36 were years of education (p = 0.022) and physical activity (p = 0.026).

**Table 3:** Multiple Linear Regression Model on PCS-36 (Physical component summary)

**Model**	**B**	**SE**	**Beta**	**95% confidence interval**	**p-value**
(Costant)	72.259	10.089		52.337; 92.182	0.000
Age in years	−0.309	0.114	−0.191	−0.534; −0.084	**0.007**
Gender (male)	1.397	1.634	0.059	−1.829; −4.624	0.394
BMI	−0.368	0.108	−0.228	−0.581; −0.155	**0.001**
Years of education	0.395	0.170	0.163	0.059; 0.731	**0.022**
PASE total score	0.031	0.014	0.153	0.004; 0.058	**0.026**
HADS anxiety	−0.338	0.246	−0.118	−0.824; 0.148	0.171
HADS depression	−0.595	0.225	−0.233	−1.040; −0.149	**0.009**
N. of medications	−0.727	0.597	−0.135	−1.906; 0.451	0.225
N. of diseases	0.449	0.788	0.062	−1.107; 2.004	0.570

Note: dependent variable: PCS-36; R^2^ = 0.291; F test = 8.840, p < 0.001.

The same model was calculated using each time one of the four physical component subscales (Physical functioning, Role-physical, Bodily pain, General health) as dependent variable. BMI was negatively associated with all the subscales (p < 0.05). Both anxiety and depression were negatively associated with Bodily pain and General Health subscales (p < 0.001), while physical activity was significant only for Physical functioning (p = 0.042). The number of medications was found negatively associated with Role-physical subscale (p = 0.01).

## Discussion

The aim of this paper was to analyse the correlates of health related quality of life (HRQL) in obese, overweight and normal weight Italians older adults. Moreover, the relationship among obesity and psychological, socio-demographic aspects was identified. Differences between the obese group and the other two groups emerged.

As our results show, the waist and hip circumferences of obese subjects were significantly higher than those in the other two groups. Unsurprisingly, the prevalence of cardiovascular diseases was significantly higher in obese subjects, in accordance with other studies [33].

Self-evaluation of perceived health status differed among the groups. Indeed, the obese group “fair/poor” rating was significantly higher than that of their overweight and normal weight counterparts. This result is consistent with other studies on elderly people in different countries [34]. Moreover, a comparison of normative data on a representative Italian population [28] demonstrates that the evaluation of the SF-36 scores obtained from our obese sample is lower, and probably consistent with the presence of pathologies linked to obesity which could impair quality of life.

Consistent with another Italian study [35], the negative impact on quality of life was observed in domains reflecting physical status, with no significant impairment in mental health. In particular, physical functioning, bodily pain, general health, vitality, role-emotional are the components of SF-36 which differed significantly among the groups.

One interesting result regards the correlation analysis done to evaluate the variables associated with PCS-36 in the total sample. We identified independent variables associated with physical health of the quality of life component (PCS-36).

Results showed that age, BMI, educational level, physical activity, depression, were significant correlates for quality of life. In this context, the role of socio-economic differences in perceived health status have been well documented [36]. Some authors identified significant differences in HRQOL by socio-demographic characteristics and behavioural risk factors, with both lower scores reported by females and less educated subjects [37]. Previous studies indicated that individuals with lower education have a poorer self perceived health status, due to several factors [38]. A possible reason may be cultural differences in values and reference levels, rather than true differences in health status [39]. Another reasons could be due to the presence of chronic diseases: some findings indicated that subjects which suffer from more than one chronic condition reported significantly lower HRQOL and the decrements were larger in PCS than in MCS [37,40].

Moreover, it exists a relationship between quality of life and age. In a recent study, a lower PCS value was reported by older patients [37]. Additionally, obesity is related to increased risk of many chronic diseases that are highly prevalent among older adults [13].

Obesity in elderly people is a decisive factor adversely affecting the health related quality of life and psychological mood status [19]. Consistent with our findings, other authors have found that subjects with a high BMI had an increased adjusted risk of developing depression compared with subjects with a normal BMI [35,41].

Our study has some limitations too. In particular, the small sample size of participants recruited exclusively in an Endocrinology Division was its main limitation with respect to representativeness and generalization of the results. Nevertheless, the endocrine disorders met in this Division, such as diabetes and thyroid diseases, are common disorders in older adults and elderly with a negative impact of quality of life [42]. Therefore, we think that there is a need to identify important characteristics related to quality of life in the older adults in order to prevent negative health outcomes, such as obesity. This is particularly true in Italy as well as in developed countries, where the prevalence of overweight and obesity is high and is increasing in elderly [43,44]. Even though a very recent paper reports that false myths and unfounded scientific beliefs exist regarding obesity in both the literature and the popular press [45], we suggest that research funding should be channelled towards studying the benefits of reducing obesity in the elderly, as also evidenced by other authors [46]. Our results indicated that some psychological aspects represent correlates of perceived health related quality of life in older adults and elderly subjects. So, some prevention programmes should be implemented for improving health in aging. Some authors found that weight loss had some benefits on postural balance and on reduction of falls of older individuals, with a positive influence on health related quality of life in older and middle age obese subjects [47-49].

Within some prevention programmes, specific personalized physical activity has to be mainly foreseen and included taking into account that physical activity is an excellent tool to prevent cardiovascular diseases, diabetes type II and also cancer in ageing as well as in obesity [50-53]. It is known that age and BMI also negatively affected engagement in physical activity [54]. In our previous study, we found that obese subjects tended to engage in physical activity significantly less than the non–obese [10]. Therefore, the physical activity and, more in general, correct life style conditions (for instance, the nutrition) have beneficial effects in reducing the inflammatory state [55,56] and in restoring the altered neuroendocrine pathway in ageing and obesity [57] with subsequent significant positive effects on the anxiety and depression [58].

## Conclusions

In conclusion, our paper showing the close negative interrelationships among some psychological factors, BMI, physical activity in obesity, offers a valid tool in order to prevent adverse effects and cardiovascular complications in old obese subjects without further pharmacological interventions due to the possible presence of various co-morbidities, such as sarcopenia, metabolic syndrome, osteoarthritis, pulmonary complications and obstructive sleep apnea syndrome (OSAS) [59]. Previously, we found that older subjects who perform regular exercise (classified as ≥ 1 h/week) had a better psychological conditions, useful for the prevention of many chronic and age-associated disorders [54]. In view of the consequences of obesity in older persons, the ESWGOP committee members are seeking answer about what is the role of physical activity in prevention and treatment of sarcopenia in older people and what exercises are most effective for older people [60].

## Competing interests

The authors declare that they have no competing interests.

## Authors’ contributions

CG participated in the study conception, interpretation of the results, preparation of manuscript. RP participated in the study design, performed statistical analysis, participated in interpretation of the results and preparation of the manuscript. RB, CG (Cristina Gagliardi) and FM participated in the study conception. EF (Elisa Felici) participated in data collection and quality check. EM participated in the study conception and revised the manuscript. MB, EF (Emanuela Faloia) and MD contributed to data collection and interpretation of results. GT contributed to study design, interpretation of results, collection of data and critical revision of the manuscript. All authors read and approved the final manuscript.

## Pre-publication history

The pre-publication history for this paper can be accessed here:

http://www.biomedcentral.com/1471-2458/14/35/prepub
